# The relationship between objective measures of physical function and serum lactate dehydrogenase in older adults with cancer prior to treatment

**DOI:** 10.1371/journal.pone.0275782

**Published:** 2022-10-06

**Authors:** Efthymios Papadopoulos, Daniel Santa Mina, Ali Abu Helal, Shabbir M. H. Alibhai

**Affiliations:** 1 Department of Medicine, University Health Network, Toronto, Ontario, Canada; 2 Faculty of Kinesiology and Physical Education, University of Toronto, Toronto, Ontario, Canada; 3 Faculty of Medicine, University of Toronto, Toronto, Ontario, Canada; 4 Institute of Health Policy, Management and Evaluation, University of Toronto, Ontario, Canada; Texas Tech University Health Science, Lubbock, UNITED STATES

## Abstract

**Background:**

Lactate dehydrogenase (LDH) reflects tumor burden and is a prognosticator of all-cause mortality in patients with cancer. Objective measures of physical function are associated with clinically relevant outcomes in older adults with cancer. However, whether physical function is associated with LDH in geriatric oncology is unknown. The objective of this study was to assess the relationship between objective measures of physical function and serum LDH in older adults with cancer prior to treatment.

**Methods:**

Data from older adults with cancer prior to treatment were retrieved from an institutional database and medical records within a tertiary cancer centre. Physical function measures involved muscle strength and physical performance. Muscle strength and physical performance were assessed through grip strength and the Short Physical Performance Battery (SPPB), respectively. LDH was log transformed using the natural logarithm. Multivariable logistic regression was used to examine the relationship between objective measures of physical function and LDH prior to treatment in all participants. Stratified analyses were performed for participants with solid and hematological cancers.

**Results:**

A total of 257 participants (mean age: 80.2y) were included in the analysis. Most participants were females (50.6%) and were diagnosed with locally advanced (26.8%), gastrointestinal disease (35.0%). The multivariable analysis indicated that SPPB was inversely associated with LDH in all participants (B = -0.019, 95%CI = -0.036 to -0.002, p = 0.028). Notably, the inverse relationship between SPPB and LDH persisted only in patients with hematological malignancies in the multivariable model of the stratified analysis (B = -0.049, 95%CI = -0.087 to -0.011, p = 0.013). Neither grip strength alone nor the combination of low grip strength and/or SPPB were associated with LDH. Compared to participants with metastatic disease, those with localized or locally advanced disease had lower serum LDH.

**Conclusion:**

Physical performance is inversely associated with serum LDH in older adults with hematological cancers prior to treatment.

## Introduction

A common metabolic characteristic of malignant cells is their ability to uptake glucose and produce large amounts of lactic acid through enhanced glycolysis, despite oxygen availability [1]. Unlike normal cells, cancer cells exhibit potent glycolytic and poor oxidative capacities as a result of metabolic dysregulation implicating poor mitochondrial function and transcription factors, such as hypoxia-inducible-factor-1 (HIF-1), c-Myc, and p53 [2]. Energy provision through glycolysis comprises the catabolism of glucose to pyruvate and subsequently, the conversion of pyruvate to lactate *regardless of normoxia*, a process that occurs in cancerous cells but not in normal cells [3].

Lactate dehydrogenase (LDH) is a potent glycolytic enzyme that is responsible for the reduction of pyruvate to lactate [4]. LDH is overexpressed in the bloodstream in response to cellular and tissue injury [5] and has diagnostic value in pathological conditions, such as liver failure [6] and acute myocardial infarction [7]. The glycolytic profile of tumors has made LDH an appealing biomarker in oncology with prognostic value [8]. Meta-analytic data suggest that LDH is significantly associated with poor overall and disease-specific survival in patients with prostate cancer [9], hepatocellular cancer [10], head and neck cancer [11], breast cancer [12], osteosarcoma [13], lung cancer [14], and some hematological malignancies [15, 16]. The prognostic value of LDH along with its role in regulating tumor stroma interaction and nutrient exchange [17] have urged the importance of considering pre-treatment LDH levels in treatment decision making [18, 19] and stimulated research in targeting LDH pharmacologically to improve disease outcomes [20]. Given the key role of LDH in tumor metabolism and mortality, it is important to explore and identify factors that may be implicated in LDH expression and subsequently develop strategies that can normalize LDH levels.

Meta-analytic data suggest that objective measures of physical function, such as muscle strength and physical performance predict overall mortality in older adults with cancer [21, 22]. The mechanisms explaining the impact of physical function on mortality in oncology remain obscure but may implicate negative health effects of physical inactivity [23] that deteriorate geriatric syndromes, such as sarcopenia [24] and frailty [25] both of which are predictors of overall mortality [26, 27]. Therefore, improving physical function through exercise is important for reducing the risk of adverse outcomes in older adults with cancer [21, 28].

Exercise and improvements in physical function may also alter LDH kinetics. In the context of tumor glycolysis, preclinical evidence suggests that endurance training decreases expression of monocarboxylate transporter 1 (MCT1) and LDH-A while concomitantly increases LDH-B in breast tumors [29]. These findings have clinical relevance given the role of MCT1 in lactate efflux and cancer progression [30], in addition to the high affinity of LDH-A and LDH-B for pyruvate and lactate, respectively [30, 31]. Although evidence on the relationship between exercise and LDH is lacking in patients with cancer, findings from a cohort study suggest that physically active older adults exhibit significantly lower serum LDH compared to their moderately active and physically inactive counterparts [32]. In agreement with these results, evidence from a cohort study of 4,006 individuals with metabolic syndrome (age range: 40–90 years) demonstrated that low physical activity and measures of poor physical function, such as weakness and slowness, were independent predictors of serum LDH [33].

Whether an inverse relationship exists between measures of physical function and LDH in older adults with cancer prior to treatment is poorly understood. Examining this relationship will enhance the understanding of modifiable factors that may be implicated in LDH overexpression and inform the need for supportive care interventions for high-risk patients. Our objective was to examine the relationship between objective measures of physical function and serum LDH in older adults with cancer prior to treatment.

## Materials and methods

This retrospective cohort study was conducted using data of older adults with cancer within the Princess Margaret Cancer Centre. Study data were retrieved from an institutional database and medical records. The database was developed concurrently with the inception of the Older Adults with Cancer Clinic (OACC) within the cancer centre in June 2015 and captures patients’ clinical characteristics to inform clinical programming and improve patient care. Patient characteristics that were retrieved from the database for the purposes of this study included: age; disease site; treatment intent; and results on eight geriatric assessment domains as previously described [34]. Medical records were used to retrieve scores of physical fitness and serum LDH. The use of the data in this study was reviewed and approved by the institution’s Research Ethics Board (ID: 17–5667) and the requirement for informed consent was waived.

### Participants

This study included older adults with a cancer diagnosis who were seen in a geriatric oncology clinic prior to cancer treatment between June 2015 (inception of geriatric clinic) to December 31, 2021.

### Physical function

Objective measures of physical function prior to treatment included muscle strength and physical performance and were assessed by trained Clinical Nurse Specialists using grip strength and the Short Physical Performance Battery (SPPB), respectively. Grip strength was assessed twice in the dominant hand using a Jamar dynamometer and the highest grip strength score was recorded. Following assessment of grip strength, the Clinical Nurse Specialist assessed participants’ SPPB using standard procedures [35]. Grip strength is a validated test and a predictor of whole-body strength in older adults [36]. SPPB involves assessment of balance (3 components), walking speed over 4 metres, and lower extremity strength through five timed chair stands and has previously been validated in older adults [37]. The score of each SPPB component ranges from 0 to 4 based on validated cut-offs and the maximum total SPPB score is 12 points indicating best physical performance [35].

### Lactate dehydrogenase

Serum LDH levels were analyzed via standard lab procedures and were captured ≤2 months prior to geriatric assessment.

### Statistical analysis

Frequencies and proportions were used to summarize categorical data, while the mean and standard deviation were used for continuous variables. Characteristics of participants with available LDH versus those with missing LDH were compared using independent samples t-tests and chi-squared tests for continuous and categorical variables, respectively. LDH (outcome variable) was not normally distributed and was log transformed using the natural logarithm. Multivariable linear regression was used to examine the relationship between measures of physical function (grip strength and SPPB) and serum LDH in all participants. Grip strength and SPPB were used as continuous predictors while the combination between the two, defined as poor grip strength and/or SPPB was used as a categorical variable using clinically relevant thresholds. The Foundation for the National Institutes of Health (FNIH) criteria were used for low muscle strength (<26kg for men and <16kg for women) [38] while a total SPPB score of ≤9/12 points was used for low physical performance [39]. Covariates in the multivariable model were selected based on clinical judgment and a p value of <0.1 in univariate analysis. The time from LDH to assessment of physical function was used as a covariate given the heterogenous range in time between the independent and dependent variables. Stratified analyses were performed for patients with solid and hematological malignancies. In sensitivity analyses we included only participants with available LDH within 2 weeks prior to assessment of grip strength and SPPB to minimize the time window between assessments. Participants with missing LDH or grip strength and SPPB were excluded from the analysis.

## Results

**Fig 1:**
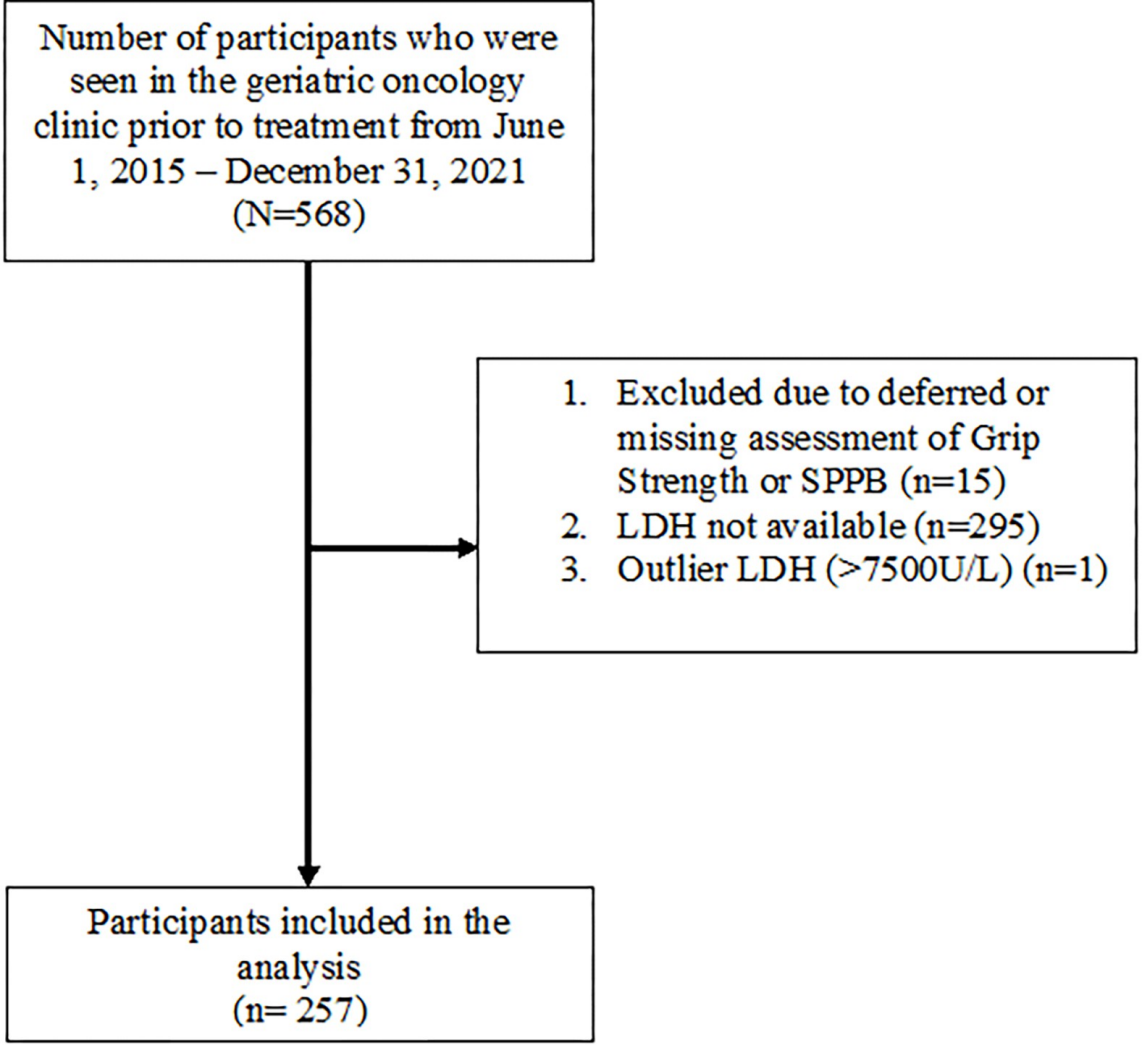
Participant flow diagram. LDH = lactate dehydrogenase; SPPB: Short Physical Performance Battery.

A total of 568 older adults prior to treatment were seen in the geriatric clinic from June 2015 to December 2021, of whom 311 were excluded from the analysis due to i) deferred assessment of grip strength and SPPB (n = 15), ii) unavailable LDH values (n = 295), and iii) high LDH value [>7500 U/L (n = 1)] likely due to hemolysis (Fig 1). The remaining participants (n = 257) who were included in the analysis had a mean age of 80.2 years (Table 1). A slight majority of our participants were females (50.6%) and were referred for cancer treatment with curative intent (60.7%). The mean grip strength and SPPB scores prior to treatment in all participants were 24.0kg and 8.1 points, respectively. Most participants (66.5%) exhibited low grip strength and/or SPPB combined. The median time and interquartile range from LDH to assessment of grip strength and SPPB was -8 days and -8 to -4 days, respectively (Table 1). Comparison of age, sex, and clinical characteristics between participants with available LDH (n = 257) and those with missing LDH (n = 295) revealed significant differences in disease stage (p<0.001) and cancer site (p<0.001) (S1 Table).

**Table 1:** Characteristics of study participants.

Characteristic	N = 257	Missing, n (%)
Age (years), mean (SD)	80.2 (6.1)	0 (0)
Sex, n (%)		0 (0)
Male	127 (49.4)	
Female	130 (50.6)	
Cancer Site, n (%)		0 (0)
Gastrointestinal	90 (35.0)	
Hematological	61 (23.7)	
Gynecological	33 (12.8)	
Head & neck	28 (10.9)	
Other^a^	25 (9.7)	
Genitourinary	20 (7.8)	
Disease stage, n (%)		0 (0)
Localized	58 (22.6)	
Locally advanced	69 (26.8)	
Hematological^b^	61 (23.7)	
Metastatic	67 (26.1)	
Unknown	2 (0.8)	
Treatment intent, n (%)		0 (0)
Curative	156 (60.7)	
Palliative	101 (39.3)	
Physical performance (grip strength and/or SPPB), n (%)		0 (0)
Normal	86 (33.5)	
Low	171 (66.5)	
Grip strength, per kg, mean (SD)	24.0 (8.9)	12 (4.7)
SPPB per point, mean (SD)	8.1 (3.0)	16 (6.2)
Time from LDH to assessment of grip strength and SPPB, days, median (IQR)	-8 (-8.0 to -4.0)	0 (0)

^a^Other cancer sites = breast (n = 12), thoracic (n = 7), skin (n = 4), other (not defined, n = 2)

IQR = interquartile range; LDH = lactate dehydrogenase; SPPB = Short Physical Performance Battery

^b^Hematological cancers are not staged further

Table 2 lists the univariate and multivariable analysis of the relationship between measures of physical function and LDH in all participants. An inverse relationship was found between pre-treatment SPPB and LDH in univariate (β = -0.022, 95%CI = -0.038 to -0.006, p = 0.008) and multivariable (β = -0.019, 95%CI = -0.036 to -0.002, p = 0.028) analyses (see Multivariable Model #1 in Table 2). No significant relationships were observed between pre-treatment grip strength and LDH in neither the univariate nor multivariable analyses. Compared with participants with normal GS and/or SPPB combined, participants with low grip strength and/or SPPB prior to treatment had higher serum LDH in univariate (β = 0.111, 95%CI = 0.010 to 0.212, p = 0.032) but not in multivariable (β = 0.087, 95%CI = -0.009 to 0.184, p = 0.076) analysis (see Multivariable Model #2 in Table 2).

**Table 2:** Linear regression models on the relationship between grip strength, SPPB, and LDH in all participants.

Variable	Univariate B (95%CI)	*p*	Multivariable model#1 B (95%CI) n = 236	*p*	Multivariable model#2 95%CI n = 256	*p*
Age, per year	-0.005 (-0.012 to 0.003)	0.25	Not used		Not used	
Grip strength, per kg	-0.002 (-0.008 to 0.003)	0.38	-0.001 (-0.007 to 0.005)	0.75	Not used	
SPPB, per point	-0.022(-0.038 to -0.006)	0.008	-0.019 (-0.036 to -0.002)	0.028	Not used	
Grip strength and/or SPPB combined			Not used			
*Low*	0.111 (0.010 to 0.212)	0.032			0.087 (-0.009 to 0.184)	0.076
*Normal*	ref.				ref.	
Sex			Not used		Not used	
*Males*	-0.052 (-0.148 to 0.044)	0.28				
*Females*	ref.					
Tx intent			Not used		Not used	
*Palliative*	0.054 (-0.044 to 0.153)	0.27				
*Curative*	ref.					
Stage						
*Localized*	-0.223 (-0.354 to -0.093)	<0.001	-0.211 (-0.348 to -0.075)	0.003	-0.206 (-0.336 to -0.075)	0.002
*Locally advanced*	-0.144 (-0.269 to -0.020)	0.024	-0.148 (-0.278 to -0.018)	0.025	-0.132 (-0.256 to -0.008)	0.037
*Hematologic*	0.120 (-0.008 to 0.249)	0.067	0.054 (-0.083 to 0.191)	0.44	0.106 (-0.022 to 0.235)	0.10
*Metastatic*	ref.		ref.		ref.	
Site^a^			Not used		Not used	
*Genitourinary*	-0.124 (-0.308 to 0.060)	0.18				
*Gynecological*	0.121 (-0.030 to 0.273)	0.11				
*Head & neck*	-0.064 (-0.224 to 0.097)	0.43				
*Hematological*	0.233 (0.109 to 0.356)	<0.001				
*Other*	-0.024 (-0.193 to 0.144)	0.77				
*Gastrointestinal*	ref.					
Time from LDH to assessment of Grip strength and SPPB (days)	0.007 (0.002 to 0.012)	0.004	0.004 (0.000 to 0.009)	0.074	0.005 (0.000 to 0.009)	0.049

Multivariable model #1 (R^2^ = 0.131) includes all participants and examines the impact of GS and SPPB on LDH separately.

Multivariable model #2 (R^2^ = 0.134) includes all participants and examines the impact of GS and/or SPPB combined on LDH.

^a^Site was not included in multivariable analyses for all participants given that participants with hematological malignancies were included in both disease stage and site. Therefore, the multivariable models include only stage which performed better in the univariate analysis whereas site was included as a covariate in the stratified analysis of participants with solid malignancies.

Note: Sample size between multivariable models differs. The multivariable model #1 includes grip strength and SPPB raw scores, whereas the multivariable model #2 includes the combination of grip strength and/or SPPB. Raw scores were extracted from medical records but were not routinely included in clinical notes. The combination of grip strength and/or SPPB which was available for all participants was extracted from the database.

In contrast to the primary analysis which included all participants regardless of cancer type, the stratified analysis for patients with solid tumors did not reveal a significant inverse relationship between pre-treatment SPPB and serum LDH in univariate (β = -0.007, 95%CI = -0.025 to 0.011, p = 0.46) and multivariable (β = -0.007, 95%CI = -0.026 to 0.012, p = 0.48) analysis (see Multivariable Model #1 in Table 3). Similarly, pre-treatment grip strength alone or the combination of low grip strength and/or SPPB were not significantly associated with LDH levels (Table 3).

**Table 3:** Linear regression models on the relationship between grip strength, SPPB, and LDH in participants with solid cancers.

Variable	Univariate B (95%CI)	*p*	Multivariable model#1 B (95%CI) n = 179	*p*	Multivariable model#2 95%CI n = 193	*p*
Age, per year	-0.003 (-0.011 to 0.005)	0.44	Not used		Not used	
Grip strength, per kg	-0.005 (-0.011 to 0.001)	0.10	0.001 (-0.008 to 0.009)	0.83	Not used	
SPPB, per point	-0.007 (-0.025 to 0.011)	0.46	-0.007 (-0.026 to 0.012)	0.48	Not used	
Grip strength and/or SPPB combined			Not used			
*Low*	0.046 (-0.057 to 0.149)	0.38			0.006 (-0.095 to 0.106)	0.91
*Normal*	ref.				ref.	
Sex						
*Males*	-0.092 (-0.189 to 0.005)	0.064	-0.064 (-0.215 to 0.087)	0.40	-0.040 (-0.147 to 0.067)	0.45
*Females*	ref.		ref.		ref.	
Tx intent						
*Palliative*	0.124 (0.026 to 0.221)	0.013	-0.083 (-0.263 to 0.097)	0.36	-0.092 (-0.261 to 0.076)	0.28
*Curative*	ref.					
Stage						
*Localized*	-0.226 (-0.344 to -0.108)	<0.001	-0.305 (-0.518 to -0.092)	0.005	-0.308 (-0.509 to -0.107)	0.003
*Locally advanced*	-0.147 (-0.260 to -0.034)	0.011	-0.189 (-0.366 to -0.013)	0.035	-0.190 (-0.356 to -0.025)	0.024
*Metastatic*	ref.		ref.		ref.	
Site						
*Genitourinary*	-0.124 (-0.290 to 0.042)	0.14	-0.107 (-0.296 to 0.083)	0.26	-0.136 (-0.301 to 0.029)	0.10
*Gynecological*	0.129 (-0.009 to 0.268)	0.067	0.034 (-0.128 to 0.196)	0.68	0.049 (-0.103 to 0.202)	0.52
*Head & neck*	-0.064 (-0.209 to 0.082)	0.39	-0.083 (-0.238 to 0.073)	0.29	-0.083 (-0.228 to 0.061)	0.25
*Other*	-0.033 (-0.187 to 0.121)	0.67	-0.041 (-0.208 to 0.127)	0.63	-0.057 (-0.211 to 0.096)	0.46
*Gastrointestinal*	ref.		ref.		ref.	
Time from LDH to assessment of Grip strength and SPPB (days)	0.006 (0.001 to 0.010)	0.022	0.004 (-0.001 to 0.009)	0.092	0.004 (0.000 to 0.009)	0.075

Multivariable model #1 (R^2^ = 0.130) includes all participants and examines the impact of GS and SPPB on LDH separately.

Multivariable model #2 (R^2^ = 0.135) includes all participants and examines the impact of GS and/or SPPB combined on LDH.

Note: Sample size between multivariable models differs. The multivariable model #1 includes grip strength and SPPB raw scores, whereas the multivariable model #2 includes the combination of grip strength and/or SPPB. Raw scores were extracted from medical records but were not routinely included in clinical notes. The combination of grip strength and/or SPPB which was available for all participants was extracted from the database.

In line with our primary analysis, a significant inverse relationship was found between pre-treatment SPPB and LDH (β = -0.049, 95%CI = -0.087 to -0.011, p = 0.013) in the stratified, multivariable analysis that included only patients with hematological cancers (Table 4). Additionally, those with low grip strength and/or SPPB combined had higher LDH levels compared to participants who had normal both grip strength and SPPB (β = 0.296, 95%CI = 0.041 to 0.552, p = 0.024) in the univariate analysis (Table 4). Among covariates in all multivariable models, localized and locally advanced stage were significantly associated with lower serum LDH compared with metastatic disease (Tables 2 and 3).

**Table 4:** Linear regression models on the relationship between grip strength, SPPB, and LDH in all participants with hematological cancers.

Variable	Univariate B (95%CI)	*p*	Multivariable model B (95%CI) n = 54	*p*
Age, per year	-0.016 (-0.037 to 0.006)	0.14	Not used	
Grip strength, per kg	0.001 (-0.012 to 0.014)	0.86	0.005 (-0.009 to 0.018)	0.49
SPPB, per point	-0.042 (-0.075 to -0.009)	0.014	-0.049 (-0.087 to -0.011)	0.013
Grip strength and/or SPPB combined			Not used	
*Low*	0.296 (0.041 to 0.552)	0.024		
*Normal*	ref.			
Sex			Not used	
*Males*	0.030 (-0.218 to 0.278)	0.81		
*Females*	ref.			
Tx intent			Not used	
*Palliative*	-0.060 (-0.33 to 0.216)	0.66		
*Curative*	ref.			
Time from LDH to assessment of Grip strength and SPPB (days)	0.008 (-0.01 to 0.027)	0.38	Not used	

Note: Participants’ stage and site were classified as hematological and therefore stage and site were not included as covariates in univariate and multivariable analyses.

Multivariable model (R^2^ = 0.118)

The inverse relationship between SPPB and serum LDH persisted in the sensitivity analyses that included participants whose LDH was assessed ≤2 weeks from assessment of objective measures of physical function (S2–S4 Tables).

## Discussion

This retrospective cohort study assessed the relationship between objective measures of physical function (grip strength and SPPB) and serum LDH in older adults with cancer prior to treatment. We found a significant inverse relationship between total SPPB scores and serum LDH in all participants regardless of cancer type. Neither grip strength alone nor combined with SPPB was associated with LDH prior to treatment. Notably, in our stratified analysis by cancer type (solid vs. hematological), the inverse relationship between SPPB and LDH persisted only in patients with hematological cancers. To further investigate the relationship between objective measures of physical function and LDH, we performed sensitivity analyses restricting our sample to those with available LDH ≤2 weeks from assessment of grip strength and SPPB. Our results of the sensitivity analyses were in line with our primary analyses. These findings are novel in oncology and warrant further investigation as the mechanisms that precipitate the potential relationship between SPPB and LDH in blood, but not solid malignancies, are elusive. It is possible that patients with hematological cancers in our study were characterized by a higher disease burden and poor physical function, whereas the absence of a significant relationship between physical function and serum LDH in patients with solid cancers was masked by including different disease stages in the stratified analysis due to the small sample. This will require larger and/or more homogeneous samples to provide additional insights.

Previous work in individuals with metabolic syndrome demonstrated that weakness and slowness were significant predictors of LDH [33]. Thus, our findings agree, in part, with the study by Chen et al. [33] given that SPPB includes assessment of gait speed. SPPB has been shown to be the strongest predictor of overall mortality in patients with cancer among other objective measures of physical function, such as grip strength, gait speed alone, and the 6-minute walking test [22], while it has also been associated with treatment complications in cancer [28]. Additionally, as proposed by the European Working Group on Sarcopenia in Older People (EWGSOP2), SPPB can be used to indicate severe sarcopenia in the presence of low muscle strength and muscle quantity or quality [40]. In this study, we demonstrated that SPPB may have additional value in oncology and particularly in older patients with hematological malignancies. The notion that improvements in physical function may co-exist with reductions in serum LDH levels has also been demonstrated in patients with chronic heart failure [41]. Specifically, patients who underwent 3 weeks of aerobic training but not controls experienced a 34.7% improvement in the 6 minute-walk test [41]. Additionally, serum from intervention but not control participants at the end of the study led to a significant reduction of LDH release from endothelial cells compared to baseline serum, indicating reduced cell apoptosis and improved endothelial health [41].

LDH levels increase in response to injury, hemolysis, myocardial infarction, and hypoxia [42]. In cancer, high LDH reflects high tumor burden, disease aggressiveness [43, 44], and may be used as a proxy marker for hypoxic gene activation in the tumor microenvironment [45].

SPPB measures physical performance which is defined as an objectively measured whole-body function related with mobility [35], involving several organs and systems that go beyond muscle strength [35]. Thus, higher LDH and low physical performance may co-exist as disease burden can negatively impact other systems and organs, but whether this is exclusive or more pronounced to blood cancers needs to be further examined.

Another area that warrants examination pertains to the potential of exercise training in normalizing LDH activity in patients with cancer. According to comprehensive reviews, acute and chronic exercise may profoundly impact the tumor microenvironment [46, 47] with studies in non-cancer populations demonstrating that specifically endurance training decreases lactate concentrations in the skeletal muscle via enhanced lactate clearance at a given workload, resulting in oxidative peripheral adaptations (e.g., mitochondrial biogenesis, increased oxidative enzymes, muscle capillarization) [4, 48]. In the context of exercise in oncology, it has been proposed that exercise training can suppress activity of LDH which in turn may reduce lactate production, thereby decreasing energy supply to the tumor [49]. In fact, preclinical evidence in cancer demonstrates that endurance training can favorably alter LDH kinetics [29]. Nonetheless, these findings require clinical translation.

The current study was cross-sectional and therefore we are unable to determine causality between the predictor and outcome variables. Another limitation is the modest sample size in the main analysis which further decreased in the stratified analysis by cancer type. However, examining the relationship between objective measures of physical function and serum LDH prior to treatment is novel in oncology and may stimulate further research given our findings on the relationship between pre-treatment physical performance and LDH in patients with hematological malignancies. Inclusion of LDH isoenzymes, particularly LDHA would have provided additional information on the relationship between LDH and physical function; however, such information was not available for participants. Thus, studies to assess the relationship between physical function and LDH isoenzymes are warranted. Additionally, given the scarcity of evidence on the relationship between objective measures of physical function and LDH in healthy older adults, we are unable to compare our findings and assess whether this relationship differs in oncology.

## Conclusion

Our findings suggest that physical performance is inversely associated with serum LDH levels in older adults with hematological cancers prior to treatment. Whether exercise training can beneficially alter LDH kinetics in patients with cancer needs to be examined.

## Supporting information

S1 Table Clinical characteristics of study participants with LDH versus those with no available LDH. (DOCX)

S2 Table Sensitivity analysis of the relationship between grip strength, SPPB, and LDH in all participants with available LDH ≤ 2 weeks prior to assessment of objective physical function. (DOCX)

S3 Table Sensitivity analysis of the relationship between grip strength, SPPB, and LDH in participants with solid cancers with available LDH ≤ 2 weeks prior to assessment of objective physical function. (DOCX)

S4 Table Sensitivity analysis of the relationship between grip strength, SPPB, and LDH in participants with hematological cancers with available LDH ≤ 2 weeks prior to assessment of objective physical function. (DOCX)

S1 Dataset (XLSX)
