# Supplementary material for: The relationship between objective measures of physical function and serum lactate dehydrogenase in older adults with cancer prior to treatment
Source: PLoS One. 2022 Oct 6;17(10):e0275782. doi: 10.1371/journal.pone.0275782 (PMC9536539; doi:10.1371/journal.pone.0275782)
Supplement: S1 Table — (DOCX) [file pone.0275782.s001.docx]

S1 Table. Clinical characteristics of study participants with LDH versus those with no available LDH.

| Characteristic | LDH available (n=257)  (Included in the analysis) | LDH unavailable  (n=295)  (Excluded from the analysis) | *P* |
| --- | --- | --- | --- |
| Age (years), mean (SD) | 80.2 (6.1) | 81.1 (7.2) | 0.12 |
| Sex, n (%) |  |  | 0.037 |
| Male | 127 (49.4) | 172 (58.3) |  |
| Female | 130 (50.6) | 123 (41.7) |  |
| Disease stage, n (%) |  |  | <0.001 |
| Localized | 58 (22.6) | 136 (46.1) |  |
| Locally advanced | 69 (26.8) | 113 (38.3) |  |
| Hematological^a^ | 61 (23.7) | 7 (2.4) |  |
| Metastatic | 67 (26.1) | 39 (13.2) |  |
| Cancer Site, n (%) |  |  | <0.001 |
| Gastrointestinal | 90 (35.0) | 77 (26.1) |  |
| Hematological | 61 (23.7) | 7 (2.4) |  |
| Gynecological | 33 (12.8) | 10 (3.4) |  |
| Head & neck | 28 (10.9) | 83 (28.1) |  |
| Other | 25 (9.7) | 46 (15.6) |  |
| Genitourinary | 20 (7.8) | 72 (24.4) |  |

^a^Hematological cancers are not staged further
