# Supplementary material for: The relationship between objective measures of physical function and serum lactate dehydrogenase in older adults with cancer prior to treatment
Source: PLoS One. 2022 Oct 6;17(10):e0275782. doi: 10.1371/journal.pone.0275782 (PMC9536539; doi:10.1371/journal.pone.0275782)
Supplement: S2 Table — (DOCX) [file pone.0275782.s002.docx]

S2 Table. Sensitivity analysis of the relationship between grip strength, SPPB, and LDH in all participants with available LDH ≤ 2 weeks prior to assessment of objective physical function.

| Variable | Univariate  B (95%CI) | *p* | Multivariable model#1  B (95%CI)  n=180 | *p* | Multivariable model#2  95%CI  n=192 | *p* |
| --- | --- | --- | --- | --- | --- | --- |
| Age, per year | -0.002 (-0.012 to 0.008) | 0.72 | Not used |  | Not used |  |
| Grip strength, per kg | -0.002 (-0.008 to 0.005) | 0.62 | 0.000 (-0.007 to 0.007) | 0.97 | Not used |  |
| SPPB, per point | -0.030 (-0.038 to -0.006) | 0.003 | -0.024  (-0.045 to -0.003) | 0.023 | Not used |  |
| Grip strength  and/or SPPB combined |  |  | Not used |  |  |  |
| *Low* | 0.144 (0.018 to 0.271) | 0.026 |  |  | 0.108 (-0.012 to 0.229) | 0.078 |
| *Normal* | ref. |  |  |  | ref. |  |
| Sex |  |  | Not used |  | Not used |  |
| *Males* | -0.036 (-0.156 to 0.084) | 0.55 |  |  |  |  |
| *Females* | ref. |  |  |  |  |  |
| Tx intent |  |  |  |  |  |  |
| *Palliative* | 0.109 (-0.013 to 0.231) | 0.079 | -0.032 (-0.204 to 0.141) | 0.71 | -0.051 (-0.220 to 0.118) | 0.55 |
| *Curative* | ref. |  |  |  |  |  |
| Stage |  |  |  |  |  |  |
| *Localized* | -0.332 (-0.497 to -0.168) | <0.001 | -0.357  (-0.594 to -0.119) | 0.003 | -0.361  (-0.597 to -0.126) | 0.003 |
| *Locally advanced* | -0.214 (-0.369 to -0.059) | 0.007 | -0.259  (-0.459 to -0.058) | 0.012 | -0.246  (-0.443 to -0.049) | 0.015 |
| *Hematologic* | 0.068 (-0.085 to 0.221) | 0.384 | -0.027 (-0.228 to 0.173) | 0.78 | 0.036 (-0.159 to 0.231) | 0.71 |
| *Metastatic* | ref. |  | ref. |  | ref. |  |
| Site^a^ |  |  | Not used |  | Not used |  |
| *Genitourinary* | -0.122 (-0.393 to 0.148) | 0.37 |  |  |  |  |
| *Gynecological* | 0.186 (0.000 to 0.373) | 0.051 |  |  |  |  |
| *Head & neck* | -0.107 (-0.329 to 0.115) | 0.34 |  |  |  |  |
| *Hematological* | 0.251 (0.105 to 0.397) | <0.001 |  |  |  |  |
| *Other* | -0.002 (-0.205 to 0.201) | 0.98 |  |  |  |  |
| *Gastrointestinal* | ref. |  |  |  |  |  |

Multivariable model #1 (R^2^= 0.161) includes all participants and examines the impact of GS and SPPB on LDH separately.

Multivariable model #2 (R^2^= 0.157) includes all participants and examines the impact of GS and/or SPPB combined on LDH.

^a^Site was not included in multivariable analyses for all participants given that participants with hematological malignancies were included in both disease stage and site. Therefore, the multivariable models include only stage which performed better in the univariate analysis whereas site was included as a covariate in the sensitivity analysis of participants with solid malignancies.

Note: Sample size between multivariable models differs. The multivariable model #1 includes grip strength and SPPB raw scores, whereas the multivariable model #2 includes the combination of grip strength and/or SPPB. Raw scores were extracted from medical records but were not routinely included in clinical notes. The combination of grip strength and/or SPPB which was available for all participants was extracted from the database.
