# Supplementary material for: The relationship between objective measures of physical function and serum lactate dehydrogenase in older adults with cancer prior to treatment
Source: PLoS One. 2022 Oct 6;17(10):e0275782. doi: 10.1371/journal.pone.0275782 (PMC9536539; doi:10.1371/journal.pone.0275782)
Supplement: S3 Table — (DOCX) [file pone.0275782.s003.docx]

S3 Table. Sensitivity analysis of the relationship between grip strength, SPPB, and LDH in participants with solid cancers with available LDH ≤ 2 weeks prior to assessment of objective physical function.

| Variable | Univariate  B (95%CI) | *p* | Multivariable model#1  B (95%CI)  n=131 | *p* | Multivariable model#2  95%CI  n=139 | *p* |
| --- | --- | --- | --- | --- | --- | --- |
| Age, per year | 0.000 (-0.011 to 0.011) | 0.98 | Not used |  | Not used |  |
| Grip strength, per kg | -0.005 (-0.012 to 0.002) | 0.17 | -0.001 (-0.009 to 0.007) | 0.78 | Not used |  |
| SPPB, per point | -0.013 (-0.037 to 0.011) | 0.28 | -0.002 (-0.026 to 0.022) | 0.87 | Not used |  |
| Grip strength  and/or SPPB combined |  |  | Not used |  |  |  |
| *Low* | 0.082 (-0.049 to 0.213) | 0.21 |  |  | 0.004 (-0.121 to 0.129) | 0.94 |
| *Normal* | ref. |  |  |  | ref. |  |
| Sex |  |  | Not used |  | Not used |  |
| *Males* | -0.101 (-0.224 to 0.022) | 0.106 |  |  |  |  |
| *Females* | ref. |  |  |  |  |  |
| Tx intent |  |  |  |  |  |  |
| *Palliative* | 0.205 (0.085 to 0.324) | <0.001 | -0.093 (-0.310 to 0.124) | 0.39 | -0.086 (-0.294 to 0.122) | 0.41 |
| *Curative* | ref. |  |  |  |  |  |
| Stage |  |  |  |  |  |  |
| *Localized* | -0.332 (-0.477 to -0.188) | <0.001 | -0.440 (-0.704 to -0.176) | 0.001 | -0.430 (-0.685 to -0.175) | 0.001 |
| *Locally advanced* | -0.214 (-0.350 to -0.078) | 0.002 | -0.296 (-0.509 to -0.083) | 0.007 | -0.279 (-0.484 to -0.074) | 0.008 |
| *Metastatic* | ref. |  | ref. |  | ref. |  |
| Site |  |  |  |  |  |  |
| *Genitourinary* | -0.122 (-0.365 to 0.120) | 0.32 | -0.198 (-0.460 to 0.063) | 0.13 | -0.215 (-0.446 to 0.017) | 0.069 |
| *Gynecological* | 0.186 (0.019 to 0.353) | 0.029 | 0.079 (-0.097 to 0.256) | 0.37 | 0.086 (-0.077 to 0.249) | 0.29 |
| *Head & neck* | -0.107 (-0.306 to 0.092) | 0.28 | -0.196 (-0.398 to 0.006) | 0.057 | -0.189 (-0.382 to 0.004) | 0.055 |
| *Other* | -0.002 (-0.184 to 0.180) | 0.98 | -0.024 (-0.218 to 0.170) | 0.80 | -0.050 (-0.226 to 0.127) | 0.58 |
| *Gastrointestinal* | ref. |  | ref. |  | ref. |  |

Multivariable model #1 (R^2^= 0.203) includes all participants and examines the impact of GS and SPPB on LDH separately.

Multivariable model #2 (R^2^= 0.201) includes all participants and examines the impact of GS and/or SPPB combined on LDH.

Note: Sample size between multivariable models differs. The multivariable model #1 includes grip strength and SPPB raw scores, whereas the multivariable model #2 includes the combination of grip strength and/or SPPB. Raw scores were extracted from medical records but were not routinely included in clinical notes. The combination of grip strength and/or SPPB which was available for all participants was extracted from the database.
