# Supplementary material for: The relationship between objective measures of physical function and serum lactate dehydrogenase in older adults with cancer prior to treatment
Source: PLoS One. 2022 Oct 6;17(10):e0275782. doi: 10.1371/journal.pone.0275782 (PMC9536539; doi:10.1371/journal.pone.0275782)
Supplement: S4 Table — (DOCX) [file pone.0275782.s004.docx]

S4 Table. Sensitivity analysis of the relationship between grip strength, SPPB, and LDH in participants with hematological cancers with available LDH ≤ 2 weeks prior to assessment of objective physical function.

| Variable | Univariate  B (95%CI) | *p* | Multivariable model  B (95%CI)  n=48 | *p* |
| --- | --- | --- | --- | --- |
| Age, per year | -0.018 (-0.042 to 0.007) | 0.15 | Not used |  |
| Grip strength, per kg | 0.002 (-0.011 to 0.016) | 0.72 | 0.007 (-0.007 to -0.022) | 0.31 |
| SPPB, per point | -0.046 (-0.083 to -0.010) | 0.015 | -0.057 (-0.100 to -0.015) | 0.010 |
| Grip strength  and/or SPPB combined |  |  | Not used |  |
| *Low* | 0.295 (0.004 to 0.587) | 0.047 |  |  |
| *Normal* | ref. |  |  |  |
| Sex |  |  | Not used |  |
| *Males* | 0.074 (-0.205 to 0.352) | 0.59 |  |  |
| *Females* | ref. |  |  |  |
| Tx intent |  |  | Not used |  |
| *Palliative* | -0.040 (-0.356 to 0.275) | 0.79 |  |  |
| *Curative* | ref. |  |  |  |
| Time from LDH to assessment of Grip strength and SPPB (days) | 0.008 (-0.01 to 0.027) | 0.38 | Not used |  |

Note: Participants’ stage and site were classified as hematological and therefore stage and site were not included as covariates in univariate and multivariable analyses.

Multivariable model (R^2^=0.139)
